# Global Trends and Perspectives of Auriculotherapy on Pain Relief: A Bibliometric and Structured Literature Review

**DOI:** 10.1155/prm/9950178

**Published:** 2026-10-28

**Authors:** Luyao Fu, Yuanyuan Feng, Tianqi Huang, Yi Huang, Zhuoyi Li, Lingqian Chen, Shiyun Sun, Ruijie Ma, Kelin He

**Affiliations:** ^1^ The Third School of Clinical Medicine Zhejiang Chinese Medical University Hangzhou Zhejiang China zcmu.edu.cn; ^2^ Department of Acupuncture and Moxibustion The Third Affiliated Hospital of Zhejiang Chinese Medical University Hangzhou Zhejiang China zjztyy.com

**Keywords:** auriculotherapy, bibliometrics, pain, RoB 2, taVNS

## Abstract

**Background:**

Auriculotherapy has gained attention for pain relief due to its safety and cost‐effectiveness, but systematic visualization and trend analysis of relevant research remain insufficient.

**Objectives:**

To map global research trends in auriculotherapy for pain and to examine whether the expansion toward clinical and evidence‐based research has translated into a sufficiently robust randomized evidence base.

**Methods:**

We searched the Web of Science Core Collection (WoSCC) and Scopus for English‐language articles and reviews published from 2008 to December 6, 2025. Bibliometric analyses were conducted using R/bibliometrix, CiteSpace, VOSviewer, SCImago Graphica, and Microsoft Excel. A nested structured review was then performed on randomized controlled trial (RCT) reports identified within the bibliometric corpus. Clinical heterogeneity was mapped by intervention and pain category, and methodological quality was assessed using Cochrane RoB 2. Because intervention protocols, comparators, outcomes, and assessment time points were highly heterogeneous, findings were synthesized using structured comparison rather than quantitative pooling.

**Results:**

A total of 525 publications were included. Research output increased markedly over time, and keyword analyses indicated a shift from mechanistic and stimulation‐focused research toward randomized trials, systematic reviews, and evidence‐based medicine. The nested review included 90 RCT reports across eight pain categories, including 37 reports of auricular acupressure, 23 of auricular acupuncture, 28 of transcutaneous auricular vagus nerve stimulation (taVNS), and 2 of laser/cryopuncture approaches. The direction and consistency of reported analgesic findings varied by pain condition and intervention. Overall, 75.6% (68/90) of RCT reports were judged to be at high risk of bias, and substantial heterogeneity was observed in intervention parameters, comparators, outcome measures, follow‐up time points, and practitioner backgrounds.

**Conclusion:**

Bibliometric growth and increasing emphasis on RCTs have not yet translated into equally robust clinical evidence. Current findings support further investigation of auriculotherapy, particularly through intervention‐ and pain‐specific approaches, but do not justify broad efficacy claims across pain conditions. Future studies should prioritize rigorous trial design, prospective registration, credible control procedures, standardized intervention reporting, harmonized outcomes, and clinically meaningful stratification.

## 1. Introduction

Pain is an unpleasant sensory and emotional experience associated with, or resembling that associated with, actual or potential tissue damage [1, 2]. The World Health Organization (WHO) categorizes pain into acute and chronic types, the latter being not only a symptom but also defined by the International Association for the Study of Pain (IASP) as a disease whose pathogenesis involves biomedical and psychological factors such as neuroinflammation, neuroplastic changes, and psychological states [3]. According to the 2020 Global Pain Index Report (fourth edition), a third of the global population suffers from daily pain, with one‐fifth of these individuals being aged under 30 [4, 5]. Chronic pain has become the third largest health problem [6]. In the long run, this can lead to anxiety and depression and changes in cognitive function, especially working memory [7].

Pain is characterized by both high prevalence and economic cost. Although its drug treatment includes nonsteroidal anti‐inflammatory drugs, opioid analgesics, ion channel drugs, and antidepressants [8], long‐term treatment may be limited by adverse effects, tolerance, dependence, or incomplete symptom control [9–11]. In contrast, nonpharmacological treatments are recommended by the American Pain Society and the American Society of Regional Anesthesia and Pain Medicine due to their effectiveness, safety, and cost‐effectiveness [10, 12].

Auriculotherapy encompasses a range of interventions delivered at the external ear, including auricular acupressure, auricular acupuncture, electrical stimulation, and transcutaneous auricular vagus nerve stimulation (taVNS). Traditional Chinese medicine attributes therapeutic effects to stimulation of auricular points connected with systemic functions through meridian theory [13], whereas modern approaches also emphasize neuroanatomical pathways, particularly the auricular branch of the vagus nerve [14]. Mechanistic studies have explored neural reflexes, neurotransmitters, inflammatory pathways, and autonomic modulation, while clinical studies have investigated musculoskeletal pain, migraine, cancer‐related pain, dysmenorrhea, and other conditions [15–18]. With the expansion of this literature, however, questions concerning trial quality, intervention standardization, and clinical comparability have become increasingly important.

To address this gap, we combined bibliometric analysis with a nested structured review of randomized controlled trial (RCT) reports. Bibliometric analysis was used to characterize the evolution of research activity, influential contributors, and emerging topics in auriculotherapy for pain. The structured review was then used to examine whether the observed shift toward clinical and evidence‐based research was accompanied by sufficiently robust trial‐level evidence by evaluating intervention characteristics, pain categories, methodological quality, and sources of heterogeneity. Linking field‐level research trends with trial‐level evidence allowed us to identify discrepancies between research growth and evidence quality and to define more specific priorities for future clinical research.

## 2. Methods

### 2.1. Data Sources

We searched the Web of Science Core Collection (WoSCC) and Scopus through the Zhejiang Chinese Medicine University website on December 6, 2025. The search covered publications from January 1, 2008, to December 6, 2025, and was restricted to English‐language articles and reviews. Database‐specific Boolean search strings were adapted to the indexing fields of each database: Topic (TS) in WoSCC and TITLE‐ABS‐KEY in Scopus. The complete reproducible search strings and applied limits are provided in Table 1. WoSCC records were exported as plain‐text files, whereas Scopus records were exported in BibTeX and CSV formats. The combined screening process is shown in Figure 1.

**TABLE 1:** Search strategy.

	Database	Complete search string

Search query	Web of Science Core Collection (WoSCC)	TS = ((pain∗ OR irritation OR ∗ache OR sore∗ OR dolor OR rankle OR ∗algia OR ∗algesia OR neuralg∗ OR myalg∗ OR arthralg∗ OR dysmenorrh∗ OR migraine OR Low‐back‐pain OR gout∗) AND (auricul∗ OR “ear acup∗” OR “ear electro∗” OR taVNS OR tVNS OR “auricular vagus nerve stimulat∗” OR BFA OR “battlefield acupuncture” OR ((laser∗ OR cryotherap∗ OR magnet∗ OR scraping OR seed OR bloodletting OR ∗sonic OR infrared OR radiofrequency) AND (auricul∗ OR ear))))
	Scopus	TITLE‐ABS‐KEY((pain∗ OR irritation OR ∗ache OR sore∗ OR dolor OR rankle OR ∗algia OR ∗algesia OR neuralg∗ OR myalg∗ OR arthralg∗ OR dysmenorrh∗ OR migraine OR Low‐back‐pain OR gout∗) AND (auricul∗ OR “ear acup∗” OR “ear electro∗” OR taVNS OR tVNS OR “auricular vagus nerve stimulat∗” OR BFA OR “battlefield acupuncture” OR ((laser∗ OR cryotherap∗ OR magnet∗ OR scraping OR seed OR bloodletting OR ∗sonic OR infrared OR radiofrequency) AND (auricul∗ OR ear))))

Document types	Article or Review Article	

Publication date	January 1, 2008 to December 6, 2025	

Search field	WoSCC = TS; Scopus = TITLE‐ABS‐KEY	

Search time	December 6, 2025	

Langue	English	

**FIGURE 1:**
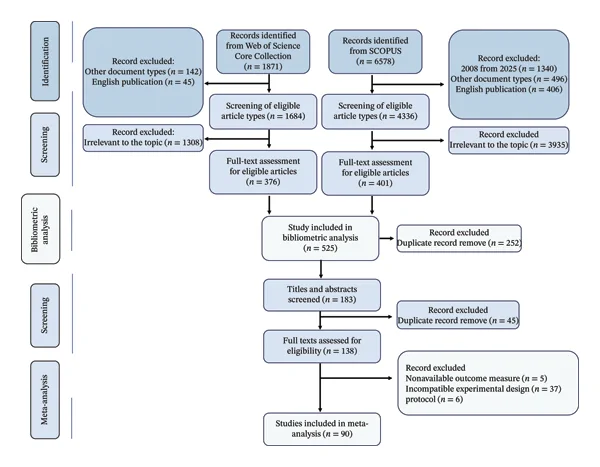
Flowchart of bibliometric screening and the nested structured review of RCT reports.

### 2.2. Visualization of Bibliometric Data

Because keyword tagging strategies and field structures differ between WoSCC and Scopus, merging keyword fields could introduce inconsistent terms and distort clustering. Therefore, keyword analyses were performed using WoSCC records only, whereas analyses of publication output, countries, institutions, authors, sources, and citations used the integrated and deduplicated dataset.

R software (v4.4.3) with the bibliometrix and openxlsx packages was used to integrate and deduplicate records. Bibliometric analyses were conducted using Biblioshiny, SCImago Graphica (v1.0.46), Microsoft Excel, CiteSpace (v6.3.1), and VOSviewer (v1.6.20). Biblioshiny was used to summarize publication characteristics, authors, countries, institutions, and sources; Excel was used to visualize annual publication trends; CiteSpace and VOSviewer were used for keyword and citation‐network analyses, and VOSviewer together with SCImago Graphica was used to visualize international collaboration.

### 2.3. Nested Structured Literature Review

#### 2.3.1. Inclusion and Exclusion Criteria

The structured review was nested within the bibliometric corpus so that field‐level trends and trial‐level evidence could be evaluated within the same literature universe. Eligible reports met the following criteria: (1) a clinical RCT design; (2) an auriculotherapy‐related intervention, including auricular acupuncture, auricular acupressure, taVNS, laser/cryopuncture, or related ear‐stimulation techniques; and (3) at least one pain‐related outcome, such as the Visual Analogu Scale (VAS), Numerical Rating Scale (NRS), Short‐Form McGill Pain Questionnaire (SF‐MPQ), analgesic consumption, or another condition‐specific pain measure. Reports were excluded if they were nonclinical studies, nonrandomized designs, reviews, conference papers, letters, animal experiments, protocols without outcome data, had nonextractable outcome information, or combined interventions in a manner that prevented attribution of the auriculotherapy component. Because this was a nested appraisal rather than an independent systematic review, the RCT component should be interpreted as a structured evaluation of randomized evidence represented in the bibliometric dataset.

#### 2.3.2. Data Organization and Analysis

The methodological quality of the included RCT reports was assessed using the Cochrane Risk of Bias 2 (RoB 2) tool [19], covering bias arising from the randomization process, deviations from intended interventions, missing outcome data, measurement of the outcome, and selection of the reported result, together with an overall judgment. Study characteristics were organized in Microsoft Excel, including population, pain condition, intervention, comparator, treatment duration, outcome measures, and publication information. Owing to major differences in auriculotherapy technique, stimulation dose, control procedures, outcome instruments, and assessment time points, a pooled meta‐analysis was not undertaken because a single average effect would have limited clinical interpretability. Instead, we performed structured evidence mapping by intervention type and pain category. Statistical significance was not treated as proof of efficacy, and all findings were interpreted alongside RoB 2 judgments and clinical heterogeneity.

## 3. Results

### 3.1. Bibliometric Analysis

#### 3.1.1. Analysis of Annual Publications

This study included 525 publications, comprising 443 articles and 82 reviews. Figure 2A shows annual and cumulative publication output. The average annual publication volume was 29.16 papers, with an annual growth rate of 18.0%. Output increased particularly after 2018, and the most recent three years each exceeded 60 publications, accounting for 35.05% of all included records. Figure 2B shows average citations per article and average citations per year. The decline in average citations for the most recent publication years should be interpreted cautiously because newer articles have had less time to accumulate citations, a well‐recognized citation‐window effect [20]. Longitudinal follow‐up would be required to determine whether the citation impact of recent work differs from earlier periods.

**FIGURE 2:**
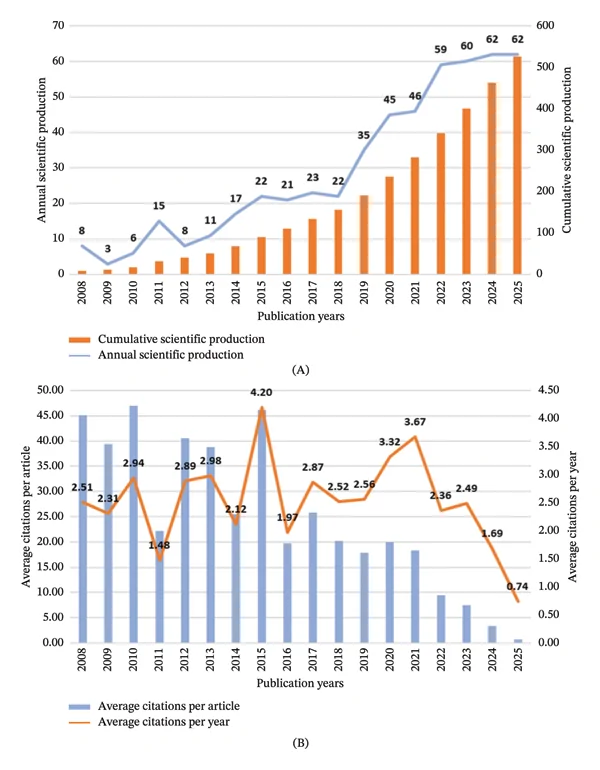
(A) Cumulative and annual scientific production and (B) average citations per article and per year.

#### 3.1.2. Analysis of Countries

Research in this field involved 34 countries. The United States (153, 29.1%), China (144, 27.4%), and Brazil (37, 7.0%) were the three most productive countries (Figure 3A and Table 2). The United States also had the largest number of multiple‐country publications (27, 17.6%), indicating a central role in international collaboration (Figure 3C). The United States and China had the highest total citation counts, whereas Germany and the United Kingdom had the highest average citations per article (Figure 3B), reflecting greater citation impact rather than necessarily higher methodological quality.

**FIGURE 3:**
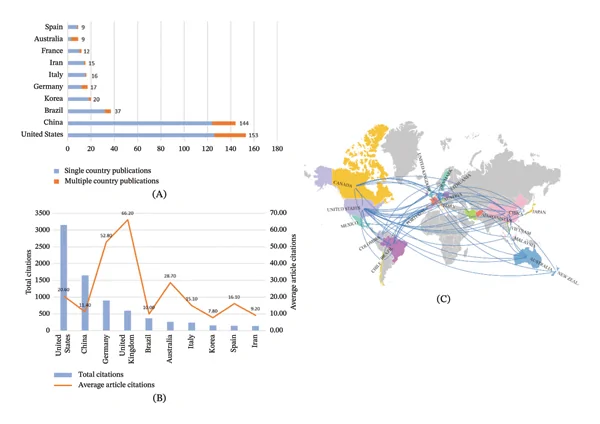
(A) Publication number for the top 10 countries; (B) total citations and average citations per country; and (C) cooperation between countries.

**TABLE 2:** Publication number and article citations for the top 10 countries.

Country	Total citations	Average article citations	Articles	Articles (%)	Single‐country publications	Multiple country publications	MCP %
United States	3147	20.60	153	29.1	126	27	17.6
China	1646	11.40	144	27.4	124	20	13.9
Germany	897	52.80	17	3.2	12	5	29.4
United Kingdom	596	66.20	9	1.7	8	1	11.1
Brazil	371	10.00	37	7	32	5	13.5
Australia	258	28.70	9	1.7	3	6	66.7
Italy	241	15.10	16	3	15	1	6.3
Korea	155	7.80	20	3.8	18	2	10
Spain	145	16.10	9	1.7	8	1	11.1
Iran	138	9.20	15	2.9	14	1	6.7

#### 3.1.3. Analysis of Institutions

A total of 736 institutions contributed to publications in this field. Figure 4A shows the 10 most productive institutions, with U.S. institutions comprising 70%. The University of Pittsburgh leads with 52 publications, followed by Johns Hopkins University (45) and Harvard University (33). Figure 4B reveals that the University of Pittsburgh was an early contributor and remains the most prolific, although its output has slowed in the past 4 years. Conversely, Johns Hopkins University, despite a later start, has demonstrated a remarkable upward trajectory in publications, signaling a robust and ongoing expansion of its research endeavors.

**FIGURE 4:**
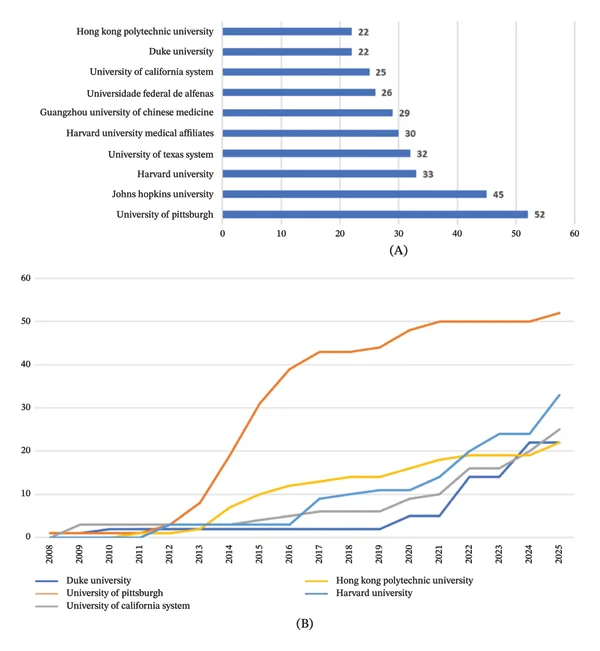
(A) Top 10 institutions’ production over time and (B) top 5 institutions’ number of publications.

#### 3.1.4. Analysis of Authors

A total of 1810 authors contributed to the literature. Figure 5 displays the publication trajectories of the 10 most productive authors. SUEN L (*n* = 16) was among the earliest contributors but has not published in this field in recent years. YEH CH was the most productive author, with 30 articles and 577 total citations, and had H‐, G‐, and M‐indices of 15, 23, and 1.071, respectively (Table 3). Articles fractionalized usually refers to the fractional count [21], which was mainly used to measure the proportion of an author’s contribution to a multiauthor paper. Among them, YEH CH had the highest apportionable count. This value represented the cumulative contribution from his co‐authored publications, equivalent to 4.77 full articles. Chien L. had the highest average citations per article (36.83), indicating substantial citation impact.

**FIGURE 5:**
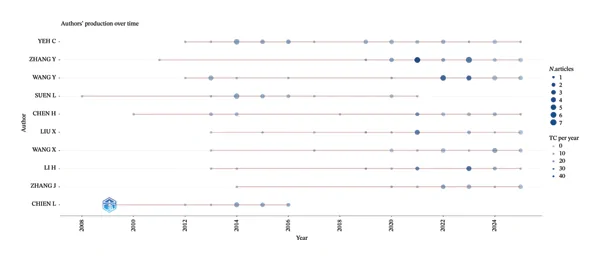
Top 10 authors’ production over time.

**TABLE 3:** Top 15 authors’ citations, index, and articles fractionalized.

Author	h_index	g_index	m_index	Total citation	Article number	Average citation count (%)	Articles fractionalized
Yeh C.	15	23	1.071	577	30	19.23	4.77
Zhang Y.	12	22	0.8	493	25	19.72	3.38
Wang Y.	11	19	0.786	400	24	16.67	3.75
Suen L.	12	16	0.667	383	16	23.94	3.08
Chen H.	8	15	0.5	255	15	17.00	2.20
Liu X.	6	15	0.462	288	15	19.20	2.53
Wang X.	4	8	0.308	82	15	5.47	2.24
Li H.	10	13	0.769	365	13	28.08	1.75
Zhang J.	5	11	0.417	127	13	9.77	1.36
Chien L.	12	12	0.706	442	12	36.83	1.94
Chen Y.	4	10	0.267	105	11	9.55	1.89
Li J.	5	9	0.385	85	11	7.73	1.64
Li X.	5	9	0.333	98	11	8.91	1.31
Li Y.	2	3	0.286	20	11	1.82	1.41
Lukkahatai N.	6	10	0.857	100	11	9.09	1.09

#### 3.1.5. Analysis of Sources

A total of 299 journals have published articles in this field. Among them, *Medical Acupuncture* (*n* = 33) is the most productive journal, followed by *Evidence-based Complementary and Alternative Medicine* (*n* = 26) and *Medicine* (*n* = 15). As shown in Figure 6A, *Evidence-based Complementary and Alternative Medicine* experienced rapid growth in its early stages but has not published any articles in this field since 2022. Additionally, according to the latest data, the journal was removed from the SCIE list on March 20, 2023, indicating potential deficiencies in article quality and ethics, although the specific reasons require further analysis and confirmation. *Medicine* has seen the fastest growth in recent years, while the publication output of other journals has generally shown a steady annual increase without any dramatic surges.

**FIGURE 6:**
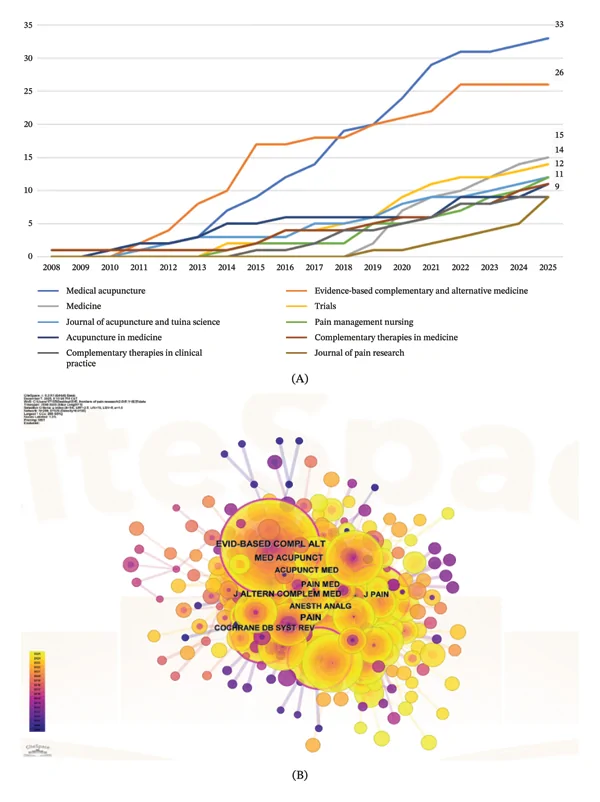
(A) Top 10 sources of production over time and (B) map of cited journals.

The cited‐journal map (Figure 6B) shows that the knowledge base spans complementary and alternative medicine as well as pain medicine. *Pain* was the most frequently cited journal. *Evidence-Based Complementary and Alternative Medicine* and *Pain Medicine* appeared among the leading sources by both citation count and centrality (Table 4), indicating prominent citation roles within the network rather than direct evidence of study quality.

**TABLE 4:** Top 5 journal counts and centrality of cited journals.

Count	Centrality	Year	Cited journals	Count	Centrality	Year	Cited journals
202	0.08	2008	PAIN	87	0.16	2008	LANCET
196	0.12	2008	**EVID-BASED COMPL ALT**	51	0.16	2008	SPINE
138	0.06	2008	J ALTERN COMPLEM MED	94	0.15	2011	J PAIN
128	0.04	2014	MED ACUPUNCT	196	0.12	2008	**EVID-BASED COMPL ALT**
122	0.11	2013	**PAIN MED**	122	0.11	2013	**PAIN MED**

#### 3.1.6. Analysis of Citation

Citation analysis helps reveal the relationships and influence of publications within a specific research field [22, 23]. Table 5 shows the top 10 citation rankings of pain for auriculotherapy materials, with 60% focusing on taVNS. The article by Butt et al. [24] in the table has been cited 370 times, with an average annual citation of 61.67. As the most cited publication, it demonstrated significant influence in the field [24]. This literature focuses on the anatomical basis of taVNS and its potential for the treatment of the nervous system and related diseases.

**TABLE 5:** Top 10 articles in total citation.

Title	DOI	Total citations	TC per year
The Anatomical Basis for Transcutaneous Auricular Vagus Nerve Stimulation	10.1111/joa.13122	370	61.67
Acupuncture and Related Techniques for Postoperative Pain: A Systematic Review of Randomized Controlled Trials	10.1093/bja/aen146	220	12.22
Treatment of Chronic Migraine with Transcutaneous Stimulation of the Auricular Branch of the Vagal nerve (Auricular t‐VNS): A Randomized, Monocentric Clinical Trial	10.1186/s10194‐015‐0543‐3	209	19.00
The Effect of Transcutaneous Vagus Nerve Stimulation on Pain Perception—An Experimental Study	10.1016/j.brs.2012.04.006	175	13.46
Evoked Pain Analgesia in Chronic Pelvic Pain Patients Using Respiratory‐Gated Auricular Vagal Afferent Nerve Stimulation	10.1111/j.1526‐4637.2012.01385.x	149	10.64
The History, Mechanism, and Clinical Application of Auricular Therapy in Traditional Chinese Medicine	10.1155/2015/495684	146	13.27
Effectiveness of Electroacupuncture or Auricular Acupuncture vs. Usual Care for Chronic Musculoskeletal Pain Among Cancer Survivors: The PEACE Randomized Clinical Trial	10.1001/jamaoncol.2021.0310	141	28.20
Auriculotherapy for Pain Management: A Systematic Review and Meta‐Analysis of Randomized Controlled Trials	10.1089/acm.2009.0451	130	8.13
Transcutaneous Auricular Vagus Nerve Stimulation Reduces Pain and Fatigue in Patients with Systemic Lupus Erythematosus: A Randomized, Double‐Blind, Sham‐Controlled Pilot Trial	10.1136/annrheumdis‐2020‐217872	112	22.40
Modulation of Brainstem Activity and Connectivity by Respiratory‐Gated Auricular Vagal Afferent Nerve Stimulation in Migraine Patients	10.1097/j.pain.0000000000000930	108	12.00

Figure 7A shows the network visualization of citation analysis, and the number of links represents the degree of association between documents. According to total link strength (TLS) and direction, citations form at least 17 distinct clusters with different colors, with limited interconnection. This may be related to the variety of diseases studied. Among them, the article by Hou et al. [25] has 53 links, suggesting that it shares common citations or thematic associations with many other articles and may have broad connections and influence in the field. This review article systematically summarizes the neuroanatomical mechanism and dominant diseases of auriculotherapy. Overall, current research still focuses on using auriculotherapy as a medium for neuromodulation to achieve analgesic effects.

**FIGURE 7:**
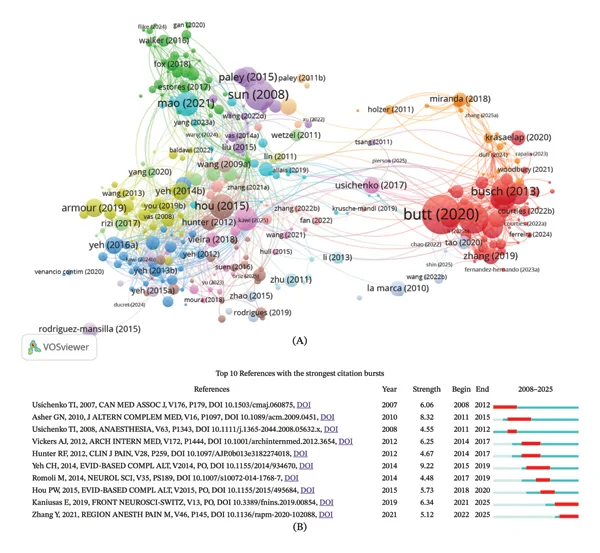
(A) Citation analysis network visualization and (B) top 10 references with the strongest citation bursts.

A citation burst refers to a period during which a publication receives a significantly higher number of citations over a minimum of 2 years [26]. Analyzing citation bursts helps identify research hotspots and predict future trends [27]. Figure 7B shows the 10 strongest reference bursts. Several burst references were systematic reviews or meta‐analyses of auriculotherapy for pain, and postoperative analgesia was another recurrent topic. The 2014 systematic review and meta‐analysis by Yeh et al. [28] had the strongest citation burst, illustrating the field’s increasing interest in evidence synthesis. Two references with ongoing bursts [29, 30] focus on taVNS, supporting its emergence as a major contemporary research direction.

#### 3.1.7. Analysis of Keywords

Keyword co‐occurrence analysis detects emerging research topics and their relations by tallying keyword frequency and co‐occurrence in literature [31]. Figure 8A visualized the keyword co‐occurrence network, where node size reflects keyword frequency and lines indicate co‐occurrence relationships. Among them, the threshold was set no less than 5 times, and 123 of the 1357 keywords met the criteria, which could be divided into 6 clusters with different colors. Key representative terms included “acupuncture,” “pain,” “auricular acupuncture,” “electrical‐stimulation,” “acupressure,” and “management.” These terms indicated that research on auriculotherapy was particularly advanced in the areas of pain and pain management, with electroacupuncture, electrical stimulation, and auricular acupuncture being important intervention methods or research directions. Table 6 listed the top 10 keywords by count and centrality, and the frequency and centrality of keywords such as “acupuncture,” “pain,” management,” “auricular acupuncture,” and “relief” were all in the top 10, indicating their significant roles in the field.

**FIGURE 8:**
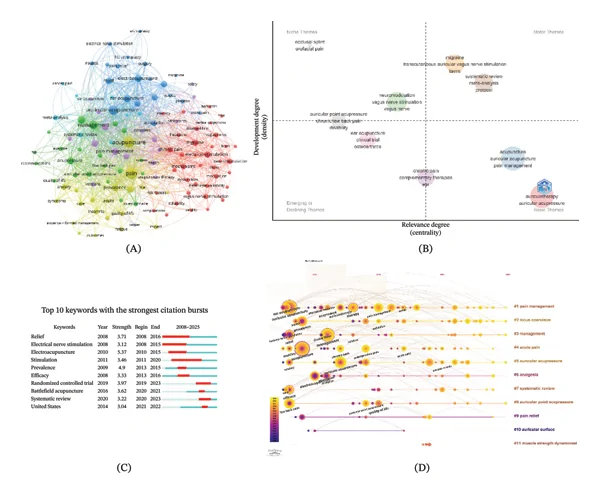
(A) Keywords co‐occurrence analysis network visualization; (B) thematic map; (C) top 10 keywords with the strongest citation bursts; and (D) timeline view map of keywords.

**TABLE 6:** Top 10 counts and centrality of keywords.

Count	Centrality	Year	Keywords	Count	Centrality	Year	Keywords
69	0.23	2009	**Acupuncture**	76	0.15	2008	**Auricular acupuncture**
67	0.22	2009	**Pain**	69	0.23	2009	**Acupuncture**
26	0.19	2008	Efficacy	67	0.22	2009	**Pain**
56	0.18	2011	**Management**	56	0.18	2011	**Management**
28	0.17	2013	Therapy	42	0.1	2008	Ear acupuncture
27	0.16	2008	Electrical stimulation	36	0.09	2012	Auricular acupressure
76	0.15	2008	**Auricular acupuncture**	32	0.08	2008	Low back pain
29	0.12	2008	**Relief**	31	0.1	2009	Prevalence
24	0.11	2020	Vagus nerve stimulation	30	0.09	2010	Electroacupuncture
18	0.11	2010	Mechanisms	29	0.12	2008	**Relief**

A thematic map is a type of map that focuses on a specific topic or phenomenon and aims to show the distribution of data in geographical space through visual elements [32]. Figure 8B was the thematic map of the author’s keywords. The basic themes, located in the lower right corner of the figure, included pain management, auricular acupressure, and auricular acupuncture. These themes had high correlation and centrality, forming the foundation and core of the research. The motor themes were located in the upper right corner, including taVNS, meta‐analysis, systematic review, and protocol. Although they were highly correlated, their development degree was relatively low, and they may be emerging research hotspots.

Citation bursts of keywords reflect the keywords that are frequently used within specific periods, revealing the evolution of research hotspots and current areas of focus. Figure 8C shows the bursts of the top 10 keywords, which can be broadly divided into two phases. The earlier phase (2008–2017) was dominated by terms such as “electrical nerve stimulation,” “stimulation,” and “efficacy,” with research primarily focused on clinical studies of auriculotherapy techniques, efficacy, and disease prevalence. The later stage (2019‐present) saw a shift toward terms like “randomized controlled trial,” and “systematic review,” highlighting the growing emphasis on evidence‐based research and the rigor of clinical methodologies.

The timeline view map depicted the temporal trends and impact of each cluster. It captured the dynamic and evolving nature of the research field by analyzing clustering, temporal distribution, and knowledge flow [33]. Figure 8D was a timeline view map generated by CiteSpace through keyword co‐occurrence network analysis. In this figure, nodes represent the first appearance of keywords, and lines indicate citation relationships. Overall, the keywords were organized into 11 distinct clusters. “Pain management” remained a persistent core theme. Mechanistic clusters such as “locus coeruleus” point to continued neurobiological investigation, while “ear acupuncture” and “electrical nerve stimulation” maintained links with later clinical themes. Frequently studied conditions included chronic pain, postoperative pain, and low back pain. The appearance of terms such as “double blind” and “adverse event” is consistent with increasing attention to trial design and safety reporting.

### 3.2. Structured Literature Review

The nested structured review included 90 RCT reports published between 2008 and 2025. Reported sample sizes ranged from 12 to 380 participants. Eight pain categories were represented: postoperative pain (32 reports), spinal pain (14), musculoskeletal pain (13), gynecological and obstetric pain (9), neuropathic pain (5), headache (4), cancer pain (2), and other pain conditions (11). Interventions comprised auricular acupressure (37 reports), auricular acupuncture (23), taVNS (28), and laser/cryopuncture approaches (2). Trial duration ranged from a single session to 12 weeks, and outcome measures included VAS, NRS, BPI, pressure pain threshold, analgesic consumption, functional measures, and condition‐specific scales. Characteristics of all 90 RCT reports are provided in Supporting Information 1.

Given the clinical and methodological discrepancies among the included studies in terms of intervention protocols, stimulation parameters, and control group design, together with high heterogeneity identified in preliminary analyses, a quantitative meta‐analysis might generate misleading average effects [34]. Accordingly, the structured review focused on the direction and consistency of findings, methodological credibility, and sources of heterogeneity rather than quantitative pooling.

#### 3.2.1. Evidence Summary by Intervention Types

Auricular acupressure was the most frequently studied intervention (37 reports). Materials included vaccaria seeds, magnetic beads, or adhesive devices applied to selected auricular points, with variable self‐ or practitioner‐administered pressure. Most reports described findings that favored auricular acupressure for at least one pain‐related outcome, but benefits were sometimes limited to selected assessment periods rather than sustained throughout follow‐up [35–39]. Control procedures were inconsistent: Some studies used adhesive tape or inert materials at the same points [40–42], whereas others used unrelated auricular points. Because pressure and tactile stimulation may themselves have nonspecific effects [43–45], the choice of sham comparator is an important source of potential bias and between‐study heterogeneity.

Auricular acupuncture was evaluated in 23 reports, including four reports of magnetic noninvasive auricular acupuncture in neonatal procedures [46–49]. Needle retention, electrical stimulation, point selection, and comparator design varied substantially. Several studies reported lower pain scores or analgesic use with auricular acupuncture [50–52], but these findings should be interpreted in the context of difficulties in participant and practitioner blinding, variable needling techniques, and inconsistent treatment schedules. These features limit direct comparison across trials and support the use of standardized reporting for acupuncture interventions.

taVNS was evaluated in 28 reports and generally targeted areas innervated by the auricular branch of the vagus nerve, particularly the cymba conchae. Sham devices without active stimulation were commonly used and allowed more credible participant blinding than was feasible for many manual auricular interventions. Reported signals included reductions in pain intensity or frequency and, in some conditions, improvement in associated symptoms such as fatigue or constipation [53, 54]. However, stimulation parameters varied widely, including frequency, pulse width, intensity, session duration, and treatment course. Few studies incorporated physiological measures of target engagement, limiting interpretation of dose–response relationships and comparability across trials.

Two reports evaluated laser or cryopuncture auriculotherapy [55, 56]. Both reported favorable pain‐related findings, but the evidence base is too small to support modality‐specific conclusions. Open‐label elements, short follow‐up, and limited replication remain important limitations.

#### 3.2.2. Evidence Summarized by Pain Types

Postoperative pain was the most frequently studied category (32 reports): 13 used auricular acupressure, 9 auricular acupuncture, 9 taVNS, and 1 cryopuncture. Findings were inconsistent. Some studies reported lower pain intensity or analgesic consumption, particularly in settings with mild‐to‐moderate surgical trauma or moderate background analgesia [57, 58], whereas approximately one‐third found no clear between‐group benefit or only transient differences [36, 37, 59–61]. Surgical type, background analgesia, timing of the intervention, and sham design likely contributed to this variability. The evidence therefore supports continued investigation of auriculotherapy as an adjunct to perioperative analgesia rather than a replacement for established multimodal pain management.

Spinal pain was represented by 14 reports, including 6 of auricular acupressure, 5 of taVNS, 2 of auricular acupuncture, and 1 of laser auriculotherapy. Most reports described findings favoring auriculotherapy for pain, disability, range of motion, or pressure pain threshold [42, 52, 62, 63]. Nevertheless, point selection, stimulation intensity, treatment duration, and comparator design were not standardized [62, 64, 65], and some studies had baseline imbalance or substantial attrition. These limitations reduce confidence in the magnitude and durability of the apparent benefit.

Musculoskeletal pain was represented by 13 reports. Auricular acupressure and taVNS each accounted for six reports, and one report used auricular acupuncture. Evidence was condition‐specific rather than uniformly positive. Auricular acupressure studies in osteoarthritis often reported pain or functional improvement, but the onset and persistence of differences varied across follow‐up periods [38, 39, 66, 67]. In contrast, two taVNS studies in fibromyalgia did not show clear analgesic benefit [54, 68]. These findings argue against treating musculoskeletal pain as a single homogeneous indication and support stratification by diagnosis and pain mechanism.

Gynecological and obstetric pain was represented by 9 reports, predominantly using auricular acupressure (7/9). Studies of dysmenorrhea and labor pain generally reported favorable pain‐related signals [17, 69, 70], whereas postpartum pain findings were inconsistent [71, 72]. Differences in pain mechanism, treatment timing, background care, compliance, and attrition may partly explain this variation. The evidence therefore suggests that clinical context and pain phenotype may be more informative than broad diagnostic grouping.

Four reports evaluated headache, predominantly migraine, with auricular acupuncture used in three. Reported findings generally favored auriculotherapy for headache intensity or frequency [73–76], but the small number of trials, limited prospective registration, and weaknesses in missing‐data handling constrain confidence in the evidence.

Five reports evaluated neuropathic pain: Three addressed cancer treatment‐related neuropathic pain [77–79] and two addressed spinal‐related neuropathic pain [80, 81]. Treatment courses were generally longer than those used for acute pain, ranging from 3 to 8 weeks. All five reports described findings favoring the intervention for at least one pain‐related outcome, but four had substantial attrition and only two reported both prospective registration and intention‐to‐treat analysis. These methodological limitations make the magnitude and reproducibility of the apparent effects uncertain.

Two reports directly addressed cancer pain [82, 83], both using auricular acupressure. Each study reported positive results, indicating that auricular acupressure effectively relieved pain intensity and breakthrough pain episodes in cancer patients. These findings suggest potential adjunctive value, but the very small evidence base and limitations in blinding, registration, and data handling preclude a firm cancer‐specific efficacy conclusion.

#### 3.2.3. Methodological Quality and Clinical Heterogeneity

RoB 2 judgments indicated limited methodological robustness across the RCT reports (Figure 9; Supporting Information 2). Eight reports (8.9%) were judged to be at low overall risk of bias, 14 (15.6%) had some concerns, and 68 (75.6%) were judged to be at high overall risk of bias. Thus, apparently favorable clinical findings should be interpreted primarily as signals requiring confirmation rather than as definitive evidence of efficacy.

**FIGURE 9:**
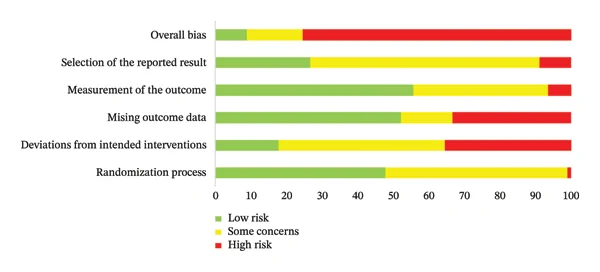
Summary of Cochrane RoB 2 judgments across the 90 included RCT reports.

Domain‐level assessment clarified the main sources of concern. For the *randomization process* (D1), 43 reports were judged low risk, 46 had some concerns, and 1 was high risk; incomplete reporting of sequence generation and allocation concealment was common. For *deviations from intended interventions* (D2), only 16 reports were low risk, whereas 42 had some concerns and 32 were high risk, reflecting the practical difficulty of blinding manual auricular interventions and the potential for performance effects. For *missing outcome data* (D3), 47 reports were low risk, 13 had some concerns, and 30 were high risk; 25 reports had attrition greater than 10%, and only 8 of these used intention‐to‐treat analysis. *Measurement of the outcome* (D4) was comparatively stronger (50 low risk, 34 some concerns, 6 high risk), although many trials relied on subjective pain scales, and assessor blinding was not universal. For *selection of the reported result* (D5), 24 reports were low risk, 58 had some concerns, and 8 were high risk. Fifty‐seven reports (63.3%) did not disclose trial registration information or a statistical analysis plan, limiting assessment of selective reporting.

Clinical heterogeneity occurred at the intervention, outcome, and population levels. Auriculotherapy included manual acupuncture, acupressure, taVNS, laser therapy, and cryopuncture, each with distinct mechanisms and delivery procedures. Even within the same modality, point selection, stimulation site, intensity, frequency, session duration, treatment course, co‐interventions, and sham design varied substantially. Outcome heterogeneity was also marked: VAS, NRS, pressure pain threshold, PIPP, BPI, McGill‐based measures, analgesic consumption, and functional outcomes were assessed at time points ranging from minutes after a single session to several weeks after treatment. Differences in pain mechanism, baseline severity, age, comorbidity, and background analgesia further limited direct comparability across reports.

Practitioner background was another source of heterogeneity. Thirty‐two reports described the interventionists’ professional background; 24 of these stated that interventionists received standardized specialized training, whereas others reported only qualifications or prior experience. Included practitioners comprised traditional Chinese medicine acupuncturists, nurses, and physicians in pain medicine or anesthesiology. Their theoretical frameworks, point‐selection strategies, and operational techniques differed, contributing to intervention variability and underscoring the need to report practitioner training and competency in future trials.

#### 3.2.4. Structured Comparison Across Pain Categories

Table 7 provides a cross‐condition summary of the randomized evidence. Reported findings were mixed in postoperative, musculoskeletal, and gynecological/obstetric pain, whereas spinal pain, headache, neuropathic pain, and direct cancer‐pain studies more often reported effects favoring auriculotherapy. However, the latter categories were represented by fewer reports and often had important methodological limitations. The “other pain” category included neonatal procedural pain, gastrointestinal pain, systemic lupus erythematosus, dialysis‐related symptoms, and other heterogeneous conditions and should not be interpreted as a single indication. This structured comparison is intended as evidence mapping rather than a pooled efficacy estimate.

**TABLE 7:** Structured comparison of randomized evidence across pain categories.

Pain category	RCT reports, n	Intervention distribution	Direction/consistency of reported findings	Key limitations/interpretation
Postoperative pain	32	Acupressure 13; acupuncture 9; taVNS 9; cryopuncture 1	Mixed; some favorable pain/analgesic findings, but approximately one‐third reported no clear between‐group benefit or only transient differences	Strong heterogeneity in surgery type, background analgesia, timing, and sham design; best interpreted as adjunctive evidence
Spinal pain	14	Acupressure 6; taVNS 5; acupuncture 2; laser 1	Predominantly favorable for pain, disability, or pressure pain threshold	Variable points/dose/course; baseline imbalance and attrition in some reports; effect magnitude and durability uncertain
Musculoskeletal pain	13	Acupressure 6; taVNS 6; acupuncture 1	Mixed and diagnosis‐dependent; osteoarthritis signals more favorable, fibromyalgia taVNS studies less consistent	Do not pool distinct diagnoses; need diagnosis‐ and mechanism‐specific trials with longer functional follow‐up
Gynecological/obstetric pain	9	Acupressure 7; taVNS 1; acupuncture 1	Mixed; dysmenorrhea/labor studies are generally more favorable than postpartum pain	Pain mechanism, timing, compliance, and attrition vary substantially
Headache	4	Acupuncture 3; taVNS 1	Generally favorable signals for intensity/frequency	Small evidence base; limited registration and missing‐data concerns
Neuropathic pain	5	Acupressure 2; taVNS 2; acupuncture 1	All reports favored intervention for at least one pain‐related outcome	High attrition in several reports; limited ITT and prospective registration; confirmatory trials needed
Cancer pain	2	Acupressure 2	Favorable adjunctive signals for pain, breakthrough pain, and symptom clusters	Only two direct cancer‐pain reports; heterogeneity in background care and methodological limitations preclude firm conclusions
Other pain conditions	11	Acupuncture 6; taVNS 4; acupressure 1	Condition‐specific favorable and null signals; not clinically homogeneous	Includes neonatal procedural pain, gastrointestinal pain, SLE, dialysis‐related symptoms, and other conditions; no single pooled interpretation is appropriate

## 4. Discussion

### 4.1. From Research Expansion to Evidence Quality: An Integrated Perspective

Bibliometric mapping showed a sustained expansion of auriculotherapy research over the past 18 years. The United States and China contributed the largest publication volumes, and seven of the 10 most productive institutions were based in the United States. This pattern may partly reflect sustained institutional investment in complementary and integrative health research [84, 85]. At the same time, citation and keyword analyses revealed an important conceptual transition: Early work emphasized electrical stimulation, mechanisms, and preliminary efficacy, whereas more recent bursts involved RCTs, systematic reviews, protocols, and pain management. On bibliometric grounds alone, the field therefore appears to be moving toward clinical translation and evidence consolidation.

Importantly, this bibliometric transition should not be interpreted as evidence that the clinical evidence base has matured to the same extent. When the bibliometric findings were examined alongside the nested structured review, a clear discrepancy emerged: Interest in RCTs and evidence synthesis increased, but 75.6% of RCT reports remained at high overall risk of bias, and substantial heterogeneity persisted across intervention, outcome, and population characteristics. Thus, growth in research quantity has not yet translated into equally robust, reproducible, and clinically comparable evidence. The next phase of the field should therefore focus less on increasing trial numbers and more on improving trial design, intervention standardization, transparent reporting, and clinically meaningful stratification.

### 4.2. Exploration in Special Populations and Complex Comorbidities

Beyond common pain diagnoses, the included literature extended to vulnerable populations and complex comorbid conditions in which treatment options may be constrained. This expansion is clinically relevant, but the breadth of populations should not be interpreted as evidence of uniform effectiveness. Instead, these studies illustrate potential settings in which minimally invasive or noninvasive auricular approaches may warrant condition‐specific evaluation.

In neonatal procedural pain, magnetic noninvasive auricular acupuncture was used in four reports [46–49]. These studies reported lower PIPP scores and smaller physiological stress responses without procedure‐related adverse events. Because neonates cannot actively participate in treatment and analgesic options are limited, noninvasive auricular stimulation is a plausible area for further research; however, replication using standardized procedures and blinded assessment remains necessary.

In older adults, reports addressed low back pain, osteoarthritis, pain in patients with cognitive impairment, and pain associated with sleep disturbance. Auricular acupressure was associated with improvements in pain and selected functional or sleep‐related outcomes in several studies [45, 86]. Its operational simplicity may be advantageous in populations with limited ability to follow complex treatment instructions, but comparative effectiveness and longer‐term safety require further study.

Perinatal studies covered labor pain, postpartum pain, and breast pain. Several reports did not show a clear analgesic advantage [41, 59, 71, 72], although some reported improvements in secondary outcomes such as depressive symptoms or pressure pain threshold [41, 87]. These mixed findings further support stratification by pain mechanism and timing rather than assuming a uniform effect across obstetric pain conditions.

Pain frequently coexists with sleep disturbance, gastrointestinal symptoms, fatigue, or immune‐related symptoms. Several included studies reported concurrent improvement in pain and associated outcomes such as sleep indices or pressure pain threshold [39, 80, 83, 86]. Such multisymptom effects are biologically plausible in the context of autonomic and neuromodulatory pathways, but they also increase the risk of selective outcome interpretation when multiple endpoints are measured. Prespecified primary outcomes and transparent analysis plans are therefore particularly important in trials of symptom clusters.

taVNS studies in gastrointestinal dysfunction and systemic lupus erythematosus reported improvements in abdominal pain, bowel symptoms, or joint‐related symptoms. Proposed mechanisms include modulation of brain–gut signaling and activation of the cholinergic anti‐inflammatory pathway [88, 89]. These mechanistic hypotheses are relevant to future trial design, but clinical validation would be strengthened by incorporating physiological markers of vagal engagement alongside prespecified patient‐centered outcomes.

Overall, studies in special populations and complex comorbidities provide signals of potential clinical utility but remain heterogeneous and frequently at risk of bias. Their main value at present is to identify specific populations and symptom clusters for targeted confirmatory research rather than to establish broad indications for auriculotherapy.

### 4.3. Cancer‐Related Pain and Integrative Oncology

Cancer‐related pain deserves separate consideration because direct tumor‐related pain and treatment‐related neuropathic pain involve different mechanisms, background treatments, and clinical goals. Only two included reports directly evaluated cancer pain [82, 83], both using auricular acupressure. One combined auricular acupressure with WHO three‐step analgesic therapy and reported lower pain intensity and fewer breakthrough‐pain episodes, whereas the other pilot study in breast cancer focused on a symptom cluster of pain, fatigue, and disturbed sleep. These studies suggest that auricular acupressure may be most appropriately investigated as an adjunct to standard oncology symptom management rather than as a substitute for established analgesic treatment.

Three additional reports addressed cancer treatment‐related neuropathic pain [77–79], including chemotherapy‐induced peripheral neuropathy and radiotherapy‐related neuropathic pain. Notably, the RELAX phase 2 randomized trial evaluated taVNS as an adjunct to pregabalin in patients with radiotherapy‐related neuropathic pain after head and neck cancer treatment [79], illustrating the growing role of neuromodulation in integrative oncology. Nevertheless, the cancer‐related evidence remains limited by small numbers of trials, heterogeneous pain mechanisms, variable background analgesia, attrition, and incomplete registration or blinding in some studies. Future oncology trials should distinguish nociceptive cancer pain from treatment‐related neuropathic pain, prespecify background analgesic regimens, and evaluate both pain intensity and clinically relevant symptom clusters while avoiding overinterpretation of multiple secondary outcomes.

### 4.4. Evidence Quality, Heterogeneity, and Priorities for Future Trials

The bibliometric and structured‐review components converge on a central finding: Research activity has expanded faster than methodological robustness. Although randomized trials, systematic reviews, and evidence‐based medicine have become prominent topics, the nested RCT appraisal showed that 75.6% of reports were at high overall risk of bias. This quantity‐quality gap explains why a large number of apparently favorable reports cannot be translated directly into high‐certainty clinical recommendations. The most recurrent threats involved incomplete randomization reporting, difficulty establishing credible blinding, missing‐data handling, lack of prospective registration or statistical analysis plans, and substantial variability in intervention delivery and outcome assessment.

The implications also differ by intervention. Manual auricular acupuncture and acupressure require more credible sham strategies and blinded outcome assessment because tactile and needling procedures make participant and practitioner blinding difficult. taVNS offers greater potential for sham‐device control, but its evidence base is limited by poorly harmonized stimulation parameters and limited verification of target engagement. Future taVNS trials should specify the stimulation site, frequency, pulse width, intensity, session duration, treatment course, device characteristics, and physiological markers when feasible. Across modalities, intervention reporting should be sufficiently detailed to allow replication.

Future RCTs should follow contemporary reporting and protocol standards, including CONSORT 2025 and SPIRIT 2025 [90, 91]. Acupuncture‐based interventions should also report the intervention rationale, needling or pressure details, treatment regimen, co‐interventions, practitioner background, and comparator procedures in line with STRICTA [92], while device‐based interventions can benefit from the broader TIDieR framework for reproducible intervention description [93]. Prospective trial registration, a publicly accessible protocol and statistical analysis plan, intention‐to‐treat analysis, sensitivity analyses for missing data, and prespecified primary outcomes should become routine. Table 8 translates the main sources of bias and heterogeneity identified in this review into practical recommendations for future studies.

**TABLE 8:** Evidence gaps and recommendations for future auriculotherapy trials.

Domain/source of heterogeneity	Observed problem in the current evidence	Potential consequence	Recommended strategy	Relevant reporting framework
Randomization and allocation concealment	Sequence generation or concealment incompletely reported in many reports	Selection bias and baseline imbalance	Use computer‐generated randomization where appropriate; conceal allocation; report sequence generation and implementation explicitly	CONSORT 2025; SPIRIT 2025
Blinding and control procedures	Manual acupuncture/acupressure make participant/practitioner blinding difficult; sham procedures varied	Performance, expectancy, and placebo‐related effects	Use credible sham controls matched for attention/tactile exposure where feasible; blind outcome assessors; report success of blinding when assessed	CONSORT 2025; STRICTA
Missing outcome data	Substantial attrition in several pain categories; limited ITT use	Attrition bias and unstable effect estimates	Plan retention strategies; perform ITT analyses; prespecify missing‐data methods and sensitivity analyses	CONSORT 2025; SPIRIT 2025
Registration and selective reporting	Many reports lacked prospective registration or an accessible SAP	Selective outcome reporting, especially in multioutcome trials	Prospectively register; publish or archive protocol/SAP; report all prespecified primary and secondary outcomes	CONSORT 2025; SPIRIT 2025
Auricular intervention specification	Point selection, materials, pressure/needling method, session dose, and treatment course varied	Poor reproducibility and between‐study comparability	Report rationale, points, stimulation method, dose, regimen, co‐interventions, and practitioner background in sufficient detail	STRICTA; TIDieR
taVNS dose and target engagement	Frequency, pulse width, intensity, site, and session duration varied widely; physiological confirmation was uncommon	Unclear dose–response relationship and uncertain biological target engagement	Standardize and fully report device/site/parameters; include physiological markers of vagal engagement when feasible	TIDieR; CONSORT 2025
Outcome selection and timing	VAS/NRS and condition‐specific scales measured at widely different time points	Difficult comparison and synthesis; multiplicity of outcomes	Prespecify a primary pain endpoint and clinically relevant time point; report MCID/responder analyses where appropriate; harmonize core outcomes	CONSORT 2025; SPIRIT 2025
Pain heterogeneity and background therapy	Distinct acute, nociceptive, neuropathic, visceral, and chronic pain conditions often discussed under one umbrella	Diluted or misleading average effects	Stratify by pain mechanism/type, clinical context, baseline severity, and background analgesia; avoid broad cross‐condition efficacy claims	Protocol‐level prespecification
Cancer‐related pain	Direct cancer pain and treatment‐related neuropathic pain differ in mechanisms and standard care	Oncology‐specific effects may be obscured or confounded by concomitant analgesia	Separate nociceptive cancer pain from treatment‐related neuropathic pain; standardize background analgesia; evaluate symptom clusters with prespecified hierarchy	CONSORT 2025; SPIRIT 2025

Clinical stratification is equally important. Auriculotherapy is an umbrella term that combines interventions with different mechanisms, doses, and implementation requirements, while pain includes mechanistically distinct conditions. Future evidence synthesis will be more informative if trials are designed and reported by intervention modality, pain mechanism or phenotype, clinical context, and background treatment. Harmonized pain outcomes and clinically relevant assessment time points would further improve comparability.

## 5. Strengths and Limitations

This study combines bibliometric mapping with a nested structured review to connect field‐level research trends with trial‐level methodological and clinical characteristics. The bibliometric component describes growth, collaboration patterns, and evolving research themes, while the structured review evaluates intervention diversity, pain‐specific findings, risk of bias, and sources of heterogeneity. This integrated design allows the apparent expansion of clinical research to be interpreted in the context of evidence quality rather than publication volume alone.

Several limitations should be acknowledged. First, the bibliometric search was restricted to English‐language publications in WoSCC and Scopus; relevant non‐English studies or records indexed only in other databases may therefore have been missed. Second, the structured review was nested within the bibliometric corpus rather than based on an independent systematic search dedicated to RCTs, which may introduce selection bias and means that the RCT component should not be interpreted as an exhaustive systematic review. Third, the structured review operated at the publication‐report level; multiple reports from the same underlying participant cohort could not always be excluded with certainty, so counts are presented as RCT reports rather than unique trials. Fourth, bibliometric indicators are sensitive to database coverage, software algorithms, author‐name disambiguation, and citation‐window effects. Fifth, substantial heterogeneity in pain conditions, intervention protocols, comparators, outcomes, and assessment time points precluded a clinically meaningful pooled meta‐analysis. Structured comparison reduces the risk of inappropriate pooling but remains descriptive and cannot provide a single summary treatment effect. Finally, emerging modalities such as laser therapy and cryopuncture were represented by very few randomized reports, limiting modality‐specific conclusions.

## 6. Conclusion

Bibliometric analysis of 525 publications showed rapid growth in auriculotherapy research and a recent shift toward randomized trials, systematic reviews, evidence‐based medicine, and taVNS. However, the nested review of 90 RCT reports revealed a clear quantity–quality gap: Three‐quarters of reports were at high overall risk of bias, and major heterogeneity was present across intervention parameters, comparators, outcome measures, time points, pain conditions, and practitioner backgrounds. Current evidence therefore supports continued investigation of auriculotherapy but does not justify broad claims of established efficacy across pain conditions. Future research should prioritize pain‐ and intervention‐specific stratification, standardized auricular and taVNS protocols, credible sham controls, prospective registration, transparent analysis, harmonized clinically meaningful outcomes, and complete intervention reporting.

## Author Contributions

Luyao Fu: conceptualization, data curation, methodology, software, visualization, writing–original draft, and writing–review and editing. Yuanyuan Feng: software, visualization, and writing–original draft. Tianqi Huang: writing–original draft. Yi Huang: data curation, software, and writing–review and editing. Zhuoyi Li: writing–review and editing. Lingqian Chen: writing–review and editing. Shiyun Sun: writing–review and editing. Kelin He: formal analysis and writing–review and editing. Ruijie Ma: funding acquisition and writing–review and editing.

## Funding

This study was supported by Key Laboratory of Acupuncture and Neurology of Zhejiang Province, Department of Neurobiology and Acupuncture Research, The Third Clinical Medical College, Zhejiang Chinese Medical University, Hangzhou, China. 2024 Zhejiang Chinese Medical University Cultivation Plan for Top Innovative Talents of Postgraduates (No. 741100G00730), Hangzhou, China. the Joint TCM Science & Technology Projects of National Demonstration Zones for Comprehensive TCM Reform (Grant No. GZY‐KJS‐ZJ‐2026‐120), Hangzhou, China.

## Disclosure

The manuscript has not been published elsewhere and is not under consideration by another journal.

## Conflicts of Interest

The authors declare no conflicts of interest.

## Supporting Information

Additional supporting information can be found online in the Supporting Information section.

## Supporting information


**Supporting Information 1** Supporting Information 1: Characteristics of the 90 included randomized controlled trials (Excel file containing study design, population, intervention parameters, and outcome measures).

**Supporting Information 2** Supporting Information 2: Cochrane RoB 2 quality assessment results for all included trials (PDF file with domain‐specific ratings and overall bias judgments).

## Data Availability

The data that support the findings of this study are available in the supporting information of this article.
