# Supplementary material for: Global Trends and Perspectives of Auriculotherapy on Pain Relief: A Bibliometric and Structured Literature Review
Source: Pain Res Manag. 2026 Oct 28;2026:9950178. doi: 10.1155/prm/9950178 (PMC13714571; doi:10.1155/prm/9950178)

	D1	D2	D3	D4	D5	Overall
Ru 2023	!	!	-	+	-	-
Gottipati 2025	-	!	+	-	-	-
Vaira 2024	+	+	-	+	-	-
Liu 2024	!	-	!	+	!	-
Asadi 2025	!	-	+	+	!	-
Vaira 2025	!	!	-	+	!	-
Gao 2024	+	-	+	!	!	-
Wetzel 2011	+	!	+	+	!	!
Zhang 2024	+	!	+	+	+	!
Kindberg 2008	+	!	+	+	!	!
Hendawy 2020	+	-	+	+	+	-
Jaic 2019	!	!	+	!	!	-
Holzer 2011	!	!	+	+	!	-
Xia 2025	+	+	+	+	+	+
Vagharseyyedin 2022	+	!	!	-	+	-
Collinsworth 2019	+	-	!	!	!	-
Zhou 2022	!	+	+	+	!	!
Patel 2022	+	!	+	+	+	!
Yin 2025	!	!	!	+	+	-
Xiong 2025	+	+	+	+	+	+
Ilfeld 2024	+	+	+	+	!	!
Ilfeld 2025	+	+	+	+	-	-
Chelly 2025	!	-	-	+	!	-
Maryam 2020	!	+	+	!	!	-
Xia 2017	+	-	+	+	!	-
Chang 2012	!	!	+	+	!	-
He 2013	!	!	+	+	!	-
Baldawi 2022	!	!	+	+	!	-
Plunkett 2018	!	-	-	-	!	-
Serritella 2021	!	-	+	-	!	-
Blank 2021	+	+	+	+	!	!
Crowell 2025	+	!	+	+	+	!
Eslami 2025	!	!	+	!	!	-
Vahedi 2021	+	-	-	!	!	-
Kwan 2013	!	!	-	!	-	-
Wang 2009	!	!	!	+	!	-
Yeh 2013	+	+	-	!	!	-
Cha 2016	+	-	-	!	-	-
Han 2024	+	!	-	!	!	-
Trinh 2024	+	!	+	+	!	!
Kim 2019	!	-	-	!	-	-
Menezes 2022	!	-	+	+	+	-
Demircioğlu 2024	!	-	-	+	!	-
Kim 2021	!	-	-	+	!	-
Lee 2018	!	!	!	+	!	-
Pourmohammadi 2025	!	!	-	!	-	-
Yeh 2013	!	-	-	!	!	-
Yeh 2015	!	!	-	+	!	-
Marignan 2014	!	!	+	+	!	-
Vas 2014	+	!	-	+	!	-
Uzlifatin 2013	!	-	+	!	!	-

+ Low risk

! Some concerns

- High risk

D1 Randomisation process

D2 Deviations from the intended interventions

D3 Missing outcome data

D4 Measurement of the outcome

D5 Selection of the reported result

Halim(1) 2023	!	!	+	!	!	-
Halim(2) 2023	!	!	-	+	!	-
Hunter 2012	+	!	!	+	!	!
Johnston 2021	+	-	+	-	+	-
Percin 2025	!	-	!	+	+	-
Kutlu 2020	!	-	-	!	!	-
Huffman 2024	+	-	-	!	!	-
Lee 2022	!	-	-	!	!	-
Lee 2023	+	-	!	!	!	-
Zhang 2022	+	!	+	!	!	-
Xia 2011	!	-	+	!	!	-
Lin 2023	!	-	!	!	!	-
Ferreira 2024	+	+	+	+	+	+
Prott 2025	+	!	+	!	+	!
Elsehrawy 2025	+	!	+	!	+	!
Bernateck 2008	+	-	!	!	!	-
Woodbury 2021	!	-	-	!	!	-
Lim 2023	!	!	-	!	!	-
Estores 2017	!	-	-	-	!	-
Jung 2025	+	+	-	+	+	-
Yang 2024	!	-	-	!	!	-
Zuo 2025	+	+	+	+	+	+
Habibabadi 2021	!	!	+	+	!	-
Allais 2011	!	-	!	+	!	-
Jin 2025	!	!	!	+	!	-
Huang 2023	+	!	-	+	+	-
Huang 2025	!	-	+	!	!	-
Yeh 2015	!	!	-	!	!	-
Li 2025	+	+	+	+	+	+
Chen 2017	+	!	-	+	!	-
Gan 2020	+	+	+	+	+	+
Kambam 2025	+	!	-	+	!	-
Moss 2015	!	!	+	!	!	-
Zhang 2022	+	!	+	+	+	!
Shi 2021	!	-	+	!	!	-
Aranow 2020	+	+	+	+	+	+
Zhang 2025	+	!	+	!	+	!
Garner 2018	+	!	+	!	+	-
Krasaelap 2019	+	+	+	+	+	+

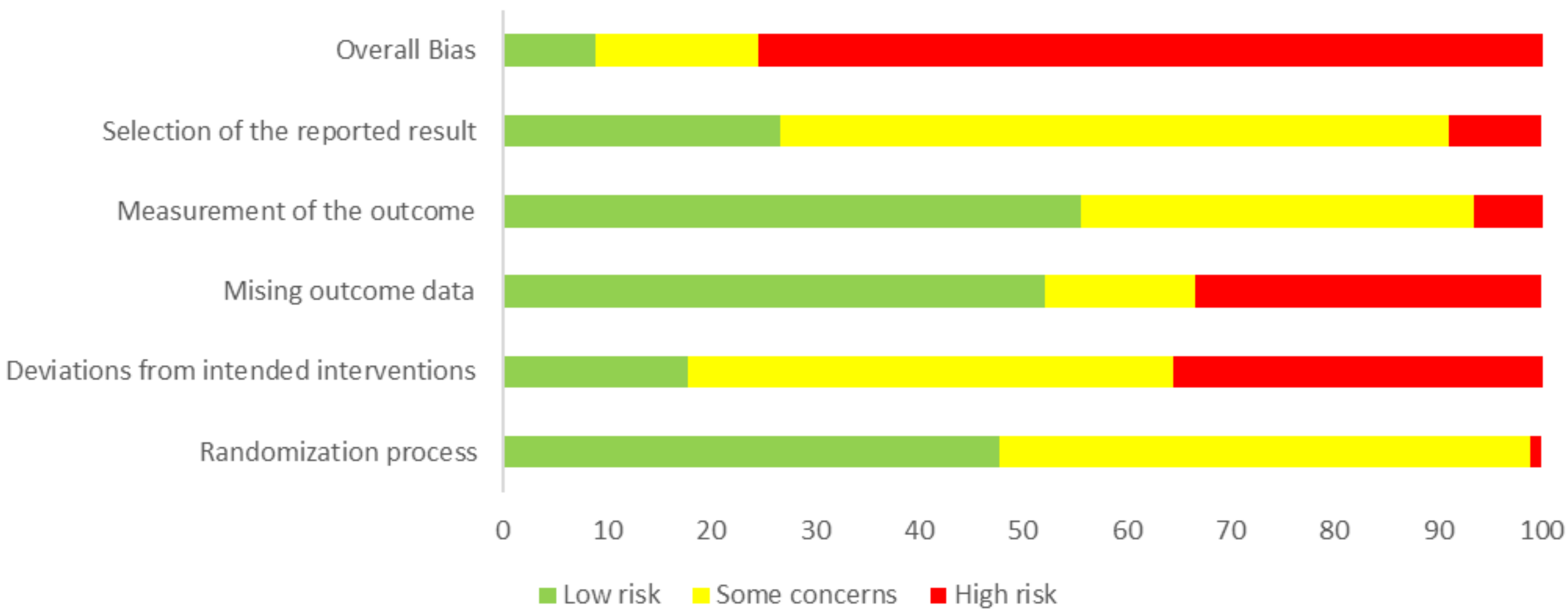

Supplement: Supplementary file 1 [file PRM-2026-9950178-s001.pdf]
